# Antiwrinkle efficacy of 1‐ethyl‐*β*‐*N*‐acetylglucosaminide, an inducer of epidermal hyaluronan production

**DOI:** 10.1111/srt.13090

**Published:** 2021-08-29

**Authors:** Yoko Endo, Hiroyuki Yoshida, Yumiko Akazawa, Kohei Yamazaki, Yukiko Ota, Tetsuya Sayo, Yoshito Takahashi

**Affiliations:** ^1^ Biological Science Research Kao Corporation Kotobuki‐cho Odawara‐shi Kanagawa Japan; ^2^ Skin Care Products Research Kao Corporation Kotobuki‐cho Odawara‐shi Kanagawa Japan

**Keywords:** 1‐ethyl‐*β*‐*N*‐acetylglucosaminide, aging, epidermis, hyaluronan, skin, wrinkles

## Abstract

**Background:**

Hyaluronan (HA) has a unique hydration capacity that contributes to firmness and bounciness of the skin. Epidermal HA declines with skin aging, which may lead to clinical signs of aging including skin wrinkles and loss of hydration and elasticity. Recently, we developed a new cosmetic agent 1‐ethyl‐*β*‐*N*‐acetylglucosaminide (*β*‐NAG2), which enhances HA production in cultured human keratinocytes. The aim of this study was to explore antiaging potential of *β*‐NAG2 in reconstructed human epidermal models and human clinical trial.

**Materials and methods:**

The amount of HA in *β*‐NAG2‐treated epidermal models by topical application was analyzed by enzyme‐linked immunosorbent assay (ELISA)‐like assay. A randomized, double‐blind and placebo‐controlled study was conducted in Japanese females (*n* = 33) by topically treating each side of the face with a lotion formulated with *β*‐NAG2 or placebo for 8 weeks.

**Results:**

Topically applied *β*‐NAG2 dose dependently increased HA production in epidermal models. Treatment with *β*‐NAG2‐formulated lotion significantly improved skin hydration and elasticity and reduced skin wrinkling in crow's foot areas when compared to the placebo formulation.

**Conclusion:**

Topically applied *β*‐NAG2 promoted epidermal HA production in vitro and showed antiwrinkle activity in vivo accompanying the improvement in skin hydration and elasticity. Our study provides a novel strategy for antiwrinkle care through *β*‐NAG2‐induced epidermal HA production.

**Abbreviations**
*β*‐NAG2: 1‐ethyl‐*β*‐*N*‐acetylglucosaminide
ELISA: enzyme‐linked immunosorbent assay
GlcUA: glucuronic acid
HA: hyaluronan
HAS: HA synthase
NAG: *N*‐acetylglucosamine
UDP: uridine diphosphate

## 1 INTRODUCTION

Skin aging is a complex biological phenomenon, which is classified into natural intrinsic aging and extrinsic aging caused by ultraviolet light. Clinically, the general features of aged skin are wrinkles, sagging, rough skin texture, and increased fragility, which is represented by decreased skin hydration and elasticity and deterioration of extracellular matrix components of the dermis.^1^, ^2^, ^3^ Previous studies suggested that reduction of hyaluronan (HA) in the papillary dermis,^4^, ^5^ and massive accumulation of abnormal elastic material and damaged and disorganized collagen fibers in the reticular dermis are associated with skin wrinkle formation.^1^, ^2^, ^6^, ^7^, ^8^ In addition to these age‐related dermal changes, skin aging is often characterized with epidermis‐related alterations such as loss of skin moisture and thinning of the epidermis.^9^, ^10^

HA, a major glycosaminoglycan, which consists of repeating disaccharide units of *N*‐acetylglucosamine (NAG) and glucuronic acid (GlcUA), is known to possess extraordinary water‐holding capacity and HA‐mediated skin hydration leads to increased firmness and bounciness.^11^ In the epidermis, high concentrations of HA are found around keratinocytes,^12^, ^13^ and other groups and we previously reported that epidermal HA plays an essential role in maintaining epidermal homeostasis and turnover by regulating cell proliferation and differentiation.^14^, ^15^, ^16^, ^17^ HA is synthesized by HA synthases (HAS1, HAS2, and HAS3), which utilize cytosolic uridine diphosphate (UDP)‐GlcUA and UDP‐NAG as substrates, and HA production in cultured human keratinocytes is mainly regulated by HAS3.^18^, ^19^, ^20^ On the other hand, epidermal HA content is reported to decline with skin aging,^21^, ^22^ which may lead to clinical signs of aging such as epidermal thinning, loss of moisture, and elasticity. Therefore, the strategies to restore the age‐related decrease in epidermal HA can be promising to remedy or prevent skin aging. Indeed, retinoids, such as all‐*trans* retinoic acid and retinol, were reported to upregulate epidermal HA production by inducing *HAS* genes in vitro and in vivo,^23^, ^24^, ^25^, ^26^ and efficiently prevent or treat signs of aging including skin wrinkles.^23^, ^27^ However, the instability of retinoids and their irritant properties limit their application in cosmetics and medicines.^23^, ^28^, ^29^ We recently developed 1‐ethyl‐*β*‐*N*‐acetylglucosaminide (*β*‐NAG2), a chemically stable NAG derivative, and demonstrated that *β*‐NAG2 significantly increases HA production in cultured normal human epidermal keratinocytes by being converted to UDP‐NAG, a precursor of HA, without affecting *HAS3* mRNA expression.^30^ In addition, since *β*‐NAG2 was found to increase the epidermal thickness by accelerating epidermal proliferation and differentiation via epidermal HA‐dependent manner in reconstructed human skin equivalents,^14^ we expect that *β*‐NAG2 could possess potential antiaging benefits.

In this study, we performed experiments in vitro and in vivo to investigate antiwrinkle effects of *β*‐NAG2. We found that topically applied *β*‐NAG2 dose dependently enhances HA production in reconstructed human epidermal models. We also found that treatment with *β*‐NAG2‐formulated lotion significantly improved skin hydration and elasticity and reduced skin wrinkling at the outer eye corner of the middle‐aged Japanese women when compared to the placebo formulation. Our data suggest that *β*‐NAG2 possesses an antiaging effect and that *β*‐NAG2‐induced epidermal HA production would be a promising strategy for antiwrinkle care.

## 2 MATERIALS AND METHODS

### 2.1 Materials

*β*‐NAG2 was provided by T. Hasegawa Co., Ltd. (Tokyo, Japan).

### 2.2 Topical application of β‐NAG2 to reconstructed human epidermal model

Reconstructed human epidermal model (EpiDerm™ EPI‐200) was purchased from MatTek Corporation (MA, USA), and cultured in EPI‐100NMM medium (MatTek Corporation, MA, USA) in a humidified atmosphere containing 5% CO_2_ at 37°C. In general, the reconstructed human epidermal model is achieved by cultivating neonatal human foreskin keratinocytes on a tissue culture insert at the air–liquid interface. It constructs a multilayer structure consisting of a fully differentiated epithelium with features of the normal human epidermis, including a stratum corneum. The gel formulations contained 10% (wt/wt) dipropylene glycol, 5% (wt/wt) ethanol, 5% (wt/wt) glycerol, 2% (wt/wt) dimethicone, 0.5% (wt/wt) polyoxyethylene hydrogenated castor oils, 0.3% (wt/wt) corboxyvinyl polymer, 0.3% (wt/wt) phenoxyethanol, 0.16% (wt/wt) potassium hydroxide, and 0.01% (wt/wt) edetate disodium in water. The test formulations without or with 0.5%, 1%, 2%, or 3.5% (wt/wt) of *β*‐NAG2 were topically applied to the epidermal models every other day for 4 days (2 μl/mm^2^).

### 2.3 Measurement of epidermal HA content

The epidermal models were incubated with 0.2 mg/ml proteinase K (ThermoFisher, MA, USA) in 0.1 M Tris‐HCl (pH 7.4), 5 mM ethylenediaminetetraacetic acid, 0.2 M NaCl, and 0.4% (wt/vol) sodium dodecyl sulfate for 2 h at 55°C. After heating for 10 min at 95°C, an equal volume of a isoamyl alcohol:phenol:chloroform (1:25:24) was added, thoroughly mixed and centrifuged to separate the aqueous phase. HA amount was determined using a sandwich‐type enzyme‐linked immunosorbent assay (ELISA)‐like assay kit (Biotech Trading Partners, CA, USA), according to the manufacturer's protocol.

### 2.4 Human clinical trial

Thirty‐three healthy Japanese female volunteers (age range, 40–58; mean age, 47.1 years) were recruited for the clinical trial. Tests were conducted in a half‐face, double‐blind manner. The test formulation containing 3.5% (wt/wt) of *β*‐NAG2 described above was topically applied on one side of the face twice daily for 8 weeks. For the control, a placebo formulation without *β*‐NAG2 was used on the opposite side of the face. Skin hydration was measured in triplicate at the corner of the eye for each subject using a Corneometer CM825 + MPA5 (Courage and Khazaka, Cologne, Germany). Measurements of skin elasticity were done in triplicate at the corner of the eye for each subject as follows: the Cutometer DUAL CT580 + MPA 580 (Courage and Khazaka, Cologne, Germany) with a 2‐mm diameter probe was used at a reduced pressure of 200 mbar with 5 s of suction followed by 2 s of release. The parameters used were U_*a*_/U_*f*_ (the overall elasticity of the skin), U_*r*_/U_*e*_ (the elastic recovery), U_*v*_/U_*e*_ (the ratio of viscosity to elasticity), and U_*r*_/U_*f*_ (the ratio of elastic recovery to the total deformation). The antiwrinkle efficacy at the eye corner was assessed by a dermatologist by visual scoring, and 3D skin replica images obtained from crow's foot areas using Silflo^®^ (Flexico Developments, Potters Bar, UK) and the 3D image analyzer Primos system (GFMesstechnik GmbH, Teltow, Germany) at 0, 4, and 8 weeks. The severity of wrinkling was graded from 7 to 0: grade 7 = very severe; grade 6 = severe; grade 5 = moderate–severe; grade 4 = moderate; grade 3 = mild–moderate; grade 2 = mild; grade 1 = none–mild; and grade 0 = none. Quarter‐intermediate grades (every 0.25) were also used in the evaluation. Before skin measurements and wrinkle scoring, each volunteer acclimated for at least 15 min prior to analysis in a room with constant humidity (40%–60%) and temperature (20–23°C). This study was approved by the Institutional Review Boards of Kao Corporation (Tokyo, Japan) and Japan Aesthetic Dermatology Symposium (Tokyo, Japan), and informed consent was obtained from all of the volunteers before the study. This clinical trial was registered at the UMIN Clinical Trials Registry as UMIN000025455.

### 2.5 Statistics

Statistical significance was assessed by Wilcoxon signed‐rank test, ANOVA, Dunnett's test and paired Student's *t*‐test using EXSUS version 8.0.0 (CAC EXICARE Corporation) or Microsoft Excel software (Office 365) (Redmond, WA, USA). A probability of *p <* 0.05 was considered significant. Nonparametric statistics (Wilcoxon signed‐rank test) and parametric statistics (paired Student's *t*‐test) were used for the data measured on an ordinal scale of measurement (skin wrinkle score) and ratio scales of measurement (skin hydration and elasticity), respectively.

## 3 RESULTS

### 3.1 Stimulatory effect of topically applied 1‐ethyl‐*β*‐*N*‐acetylglucosaminide (*β*‐NAG2) on HA production in reconstructed human epidermal model

**FIGURE 1:**
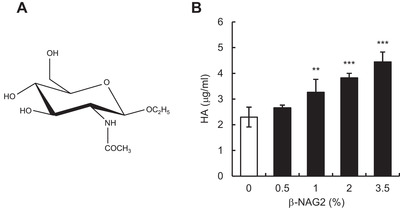
Effect of topically applied *β*‐NAG2 on HA production in reconstructed human epidermal model. (A) Chemical structure of *β*‐NAG2. (B) HA production in epidermal models topically treated with *β*‐NAG2. The epidermal models were topically treated with formulations containing *β*‐NAG2 (0%, 0.5%, 1%, 2%, or 3.5%, i.e., 0 mM, 20 mM, 40 mM, 80 mM, or 140 mM, respectively) for 4 days. HA in the epidermal models was quantified by ELISA‐like assay. Values are expressed as means ± SD (*n* = 3) and shown as μg/ml. Dunnett's test was used for statistical analysis. *β*‐NAG2, 1‐ethyl‐*β*‐*N*‐acetylglucosaminide; ELISA, enzyme‐linked immunosorbent assay; HA, hyaluronan. ***p < 0.001; **p < 0.01

Previously, we demonstrated that *β*‐NAG2 (Figure 1A) exogenously added into culture medium increases HA production in cultured normal human epidermal keratinocytes, reconstructed human skin equivalents and organ cultured skin explants.^14^, ^30^ In the present study, we examined whether topically applied *β*‐NAG2 increases HA production in reconstructed human epidermal models. The epidermal equivalents were topically treated for 4 days with gel formulations containing *β*‐NAG2 (0%, 0.5%, 1%, 2%, or 3.5%, i.e., 0, 20, 40, 80, or 140 mM, respectively), and epidermal HA content was measured by a sandwich ELISA‐like assay using HA‐binding protein. As shown in Figure 1B, *β*‐NAG2 dose dependently enhanced the amount of epidermal HA, showing the highest upregulation at a concentration of 3.5%, that is, 1.9‐fold increase in HA production. These results indicate that *β*‐NAG2 topically applied onto the skin surface of the reconstructed human epidermal models can efficiently act on keratinocytes in the epidermis to produce HA.

### 3.2 Effect of *β*‐NAG2 on skin wrinkling in crow's foot areas of Japanese females

**FIGURE 2:**
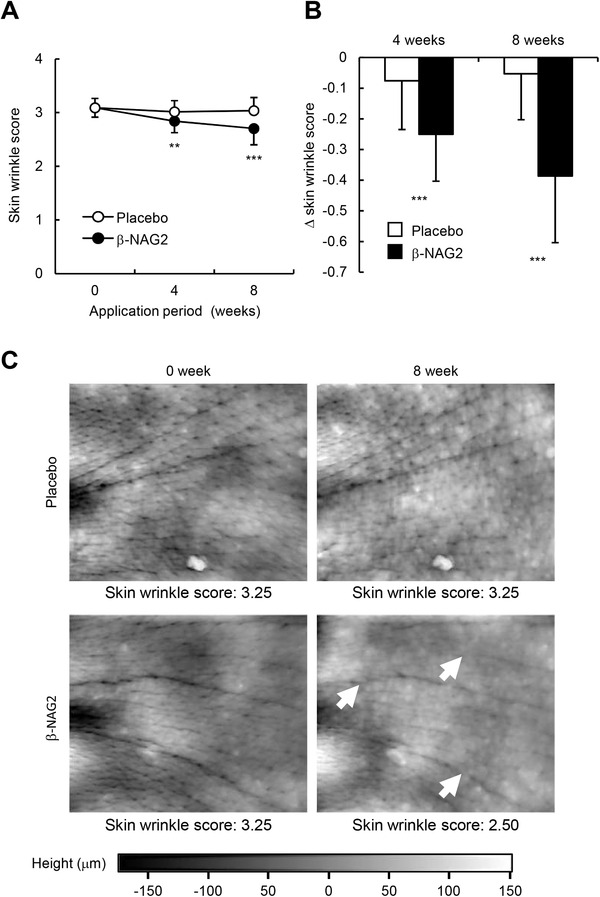
Changes in skin wrinkle score at the eye corner of the healthy Japanese females after the treatment of the placebo or the formulation containing 3.5% *β*‐NAG2 for 8 weeks. (A and B) The visual evaluation was conducted by a dermatologist via a double‐blind method. The time course of changes in skin wrinkle score (A) and extent of changes in skin wrinkle score at 4 and 8 weeks compared to 0 week (B) are shown. Values are expressed as mean ± SD (*n* = 33). The Wilcoxon signed‐rank test was used for statistical analysis when compared with placebo. ***p < 0.001; **p < 0.01. (C) Representative 3D replica images of the outer corner of the eye before (0 week) and after an 8‐week treatment with the placebo or the formulation containing 3.5% *β*‐NAG2. 3D replica image was obtained by the Primos system. Significant improvements of skin wrinkles (arrows) were observed. *β*‐NAG2, 1‐ethyl‐*β*‐*N*‐acetylglucosaminide

The potential of *β*‐NAG2 as an antiwrinkle agent was investigated in 33 healthy middle‐aged Japanese females who topically applied the test formulation containing 3.5% of *β*‐NAG2 on one side of the face and the placebo formulation without *β*‐NAG2 on the opposite side of the face twice daily for 8 weeks. As shown in Figure 2A,C and Table 1, the skin wrinkle scores at the outer eye corner, assessed by visual scoring by the dermatologist and by 3D skin replica imaging, were significantly reduced in the side treated with the *β*‐NAG2‐formulated lotion compared to the opposite side treated with the placebo formulation after 4 and 8 weeks of treatment. Changes in the skin wrinkling scores between the *β*‐NAG2‐formulated and placebo lotions were significantly different as early as after 4 weeks of treatment (Figure 2B). These results clearly revealed that *β*‐NAG2 has an antiwrinkle activity in crow's foot areas of middle‐aged Japanese females. During the clinical trial, no skin symptoms including dermatitis or allergy were observed in the volunteers.

**TABLE 1:** Skin wrinkle scores, skin hydration, and skin elasticity values

	Week	*β*‐NAG2 Mean ± SD	Placebo Mean ± SD	*p*
Skin wrinkle score	0	3.091 ± 0.175	3.091 ± 0.175	1.000
	4	2.841 ± 0.215	3.015 ± 0.207	0.003^**^
	8	2.705 ± 0.303	3.038 ± 0.243	0.000^***^
Skin hydration (au)	0	43.532 ± 12.665	44.149 ± 12.461	0.019^*^
	4	52.586 ± 6.323	49.382 ± 6.190	0.005^**^
	8	54.752 ± 7.697	48.931 ± 6.492	0.000^***^
U_*a*_/U_*f*_ value	0	0.458 ± 0.069	0.468 ± 0.054	0.375
	4	0.476 ± 0.046	0.446 ± 0.046	0.014^*^
	8	0.488 ± 0.070	0.433 ± 0.057	0.000^***^
U_*r*_/U_*e*_ value	0	0.526 ± 0.063	0.517 ± 0.062	0.413
	4	0.547 ± 0.060	0.525 ± 0.056	0.019^*^
	8	0.533 ± 0.067	0.498 ± 0.056	0.007^**^
U_*v*_/U_*e*_ value	0	0.918 ± 0.143	0.890 ± 0.166	0.323
	4	0.906 ± 0.140	0.926 ± 0.160	0.410
	8	0.808 ± 0.133	0.914 ± 0.123	0.000^***^
U_*r*_/U_*f*_ value	0	0.276 ± 0.037	0.274 ± 0.027	0.790
	4	0.288 ± 0.030	0.273 ± 0.027	0.018^*^
	8	0.297 ± 0.046	0.261 ± 0.031	0.000^***^

*Note*: Wilcoxon signed‐rank test for skin wrinkle score and paired Student's *t*‐test for skin hydration and elasticity values. Abbreviation: NAG2, 1‐ethyl‐*β*‐*N*‐acetylglucosaminide. ^***^ *p <* 0.001; ^**^ *p <* 0.01; ^*^ *p <* 0.05.

### 3.3 Effect of *β*‐NAG2 on skin hydration and elasticity in crow's foot areas of the Japanese females

**FIGURE 3:**
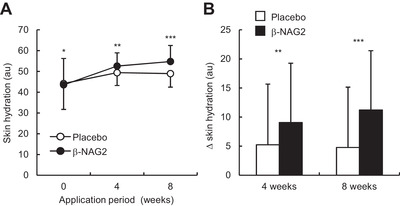
Changes in skin hydration at the eye corner of the healthy Japanese females after the treatment of the placebo or the formulation containing 3.5% *β*‐NAG2 for 8 weeks. (A and B) The time course of changes in skin hydration (A), and extent of changes in skin hydration (B) at 4 and 8 weeks compared to 0 week are shown. Values are expressed as mean ± SD (*n* = 33). Paired Student's *t*‐test was used for statistical analysis when compared with placebo. *β*‐NAG2, 1‐ethyl‐*β*‐*N*‐acetylglucosaminide. ***p < 0.001; **p < 0.01; *p < 0.05

**FIGURE 4:**
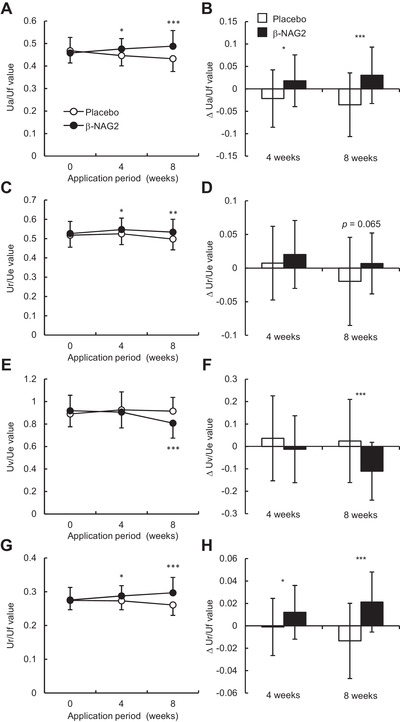
Changes in skin elasticity parameters (U_*a*_/U_*f*_, U_*r*_/U_*e*_, Uv/U_*e*_, and U_*r*_/U_*f*_) at the eye corner of the healthy Japanese females after the treatment of the placebo or the formulation containing 3.5% *β*‐NAG2 for 8 weeks. (A – H) The time course of changes in U_*a*_/U_*f*_ (A), U_*r*_/U_*e*_ (C), Uv/U_*e*_ (E), and U_*r*_/U_*f*_ (G), and the extent of changes in U_*a*_/U_*f*_ (B), U_*r*_/U_*e*_ (D), Uv/U_*e*_ (F), and U_*r*_/U_*f*_ (H) at 4 and 8 weeks compared to 0 weeks are shown. Values are expressed as mean ± SD (*n* = 33). Paired Student's *t*‐test was used for statistical analysis when compared with placebo. *β*‐NAG2, 1‐ethyl‐*β*‐*N*‐acetylglucosaminide. ***p < 0.001; **p < 0.01; *p < 0.05

Previous studies reported that skin hydration and elasticity are negatively correlated with skin roughness.^31^, ^32^ Therefore, we evaluated skin hydration and elasticity (U_*a*_/U_*f*_, the overall elasticity of the skin; U_*r*_/U_*e*_, the elastic recovery; Uv/U_*e*_, the ratio of viscosity to elasticity and U_*r*_/U_*f*_, the ratio of elastic recovery to the total deformation) at the outer eye corner of the volunteers during scheduled visits (0, 4, and 8 weeks). As shown in Figure 3A and Table 1, treatment with *β*‐NAG2‐formulated lotion significantly enhanced skin hydration value after 4 and 8 weeks of treatment compared to the placebo formulation. Changes in skin hydration values between placebo and *β*‐NAG2‐formulated lotions were found to be significant after 4 and 8 weeks of treatment (Figure 3B). In addition, treatment with *β*‐NAG2‐formulated lotion significantly enhanced U_*a*_/U_*f*_, U_*r*_/U_*e*_ and U_*r*_/U_*f*_ values after 4‐ and 8‐week treatment, and significantly reduced Uv/U_*e*_ value after 8‐week treatment when compared to the placebo formulation (Figure 4A,C,E,G and Table 1). Changes in U_*a*_/U_*f*_, U_*r*_/U_*e*_, Uv/U_*e*_, and U_*r*_/U_*f*_ values were analyzed after 4 and 8 weeks of treatment, and the differences between placebo and *β*‐NAG2‐formulated lotions were found to be significant after 4 and 8 weeks of treatment for U_*a*_/U_*f*_ and U_*r*_/U_*f*_ values, and after 8 weeks of treatment for Uv/U_*e*_ value (Figure 4B,F,H). These data suggest that *β*‐NAG2‐mediated improvement in skin hydration and elasticity may attribute to the improvement of skin wrinkle scores by the *β*‐NAG2‐formulated lotion. Taken together, these results indicate that *β*‐NAG2 is an inducer of epidermal HA production with antiwrinkle efficacy that can effectively improve hydration and elasticity in human skin.

## 4 DISCUSSION

In the present study, we have demonstrated that topically applied *β*‐NAG2 exerts a stimulatory effect on HA production in reconstructed human epidermal model. In addition, our clinical study has shown that topical treatment of facial skin with a formulation containing *β*‐NAG2 is effective in moisturizing the skin, improving elasticity and reducing wrinkles.

Our previous study revealed that *β*‐NAG2 promotes HA production in cultured normal human skin keratinocytes by increasing the intracellular pool of UDP‐NAG, a precursor of HA.^30^ In the present study, we demonstrate that topically applied *β*‐NAG2 dose dependently enhances HA production in reconstructed human epidermal models. Since our preliminary study suggested the transdermal absorption of *β*‐NAG2 (data not shown), it is tempting to speculate that *β*‐NAG2 can efficiently penetrate into the epidermis and stimulate keratinocytes to produce HA. In the previous study, we also showed that *β*‐NAG2 is converted to NAG, an endogenous compound that naturally occurs in the human body, by the action of endogenous *β*‐*N*‐acetylglucosaminidase in keratinocytes, and is finally utilized for HA production as a substrate.^30^ Thus, it can be expected that a long‐term topical application of *β*‐NAG2 on the face would not cause safety concerns. In addition, *β*‐NAG2 is chemically stable in formulations because its reducing end is structurally modified with an ethyl group to suppress the Maillard reaction with amino acid residues of compounds.^30^ From these observations, *β*‐NAG2 is considered to be a suitable inducer of epidermal HA production for cosmetics or pharmaceutical products.

HA is well known as a key molecule for skin moisture and elasticity that clinically impacts skin firmness and bounciness due to its unique capacity to bind and retain water molecules.^33^ HA is widely distributed in body tissues, and more than 50% of the total body HA content is present in the dermis and epidermis of the skin.^11^ In the epidermis, HA plays a key role in maintaining extracellular spaces to facilitate the transport of ion solutes and nutrients to cells, regulating epidermal homeostasis and turnover by controlling keratinocyte proliferation and differentiation, and preserving tissue hydration.^11^, ^14^, ^15^, ^16^, ^17^ In aged skin, one of the most drastic changes observed is the marked decrease in epidermal HA. Reductions in epidermal HA are consistently found in photoaged skin and in intrinsically aged skin,^21^, ^22^ resulting in loss of hydration and elasticity. The present study showed that treatment with *β*‐NAG2‐formulated lotion significantly improves skin hydration and elasticity (U_*a*_/U_*f*_, U_*r*_/U_*e*_, Uv/U_*e*_, and U_*r*_/U_*f*_ values), thus suggesting that *β*‐NAG2 could aid in counteracting the age‐dependent decrease in skin hydration and elasticity. In addition, we also showed that topical application of *β*‐NAG2 on human facial skin elicits a significant improvement in skin wrinkling. Since previous studies reported that U_*a*_/U_*f*_, Uv/U_*e*_, and U_*r*_/U_*f*_ values and skin hydration correlate with skin roughness,^31^, ^32^ it is plausible to speculate that enhanced HA production in keratinocytes mediated by *β*‐NAG2 may result in improvement in skin moisture and elasticity through increased water retention, bounciness, and firmness, which may in turn lead to a decrease in skin wrinkling. On the other hand, we previously reported that decrease of HA in the papillary dermis is associated with skin wrinkle formation,^4^, ^5^ and thus further studies on the effect of *β*‐NAG2 on HA production in dermal fibroblasts and HA amount in the papillary dermis are definitely required. A potential limitation of this study was that our clinical study included only middle‐aged Japanese females between the ages of 40–58. Therefore, it would be desirable for further larger scale studies to be conducted on females in different age groups, males, and/or subjects in different ethnic groups to generalize the antiwrinkle efficacy of *β*‐NAG2. It is well known that the photoaging process includes the decline of dermal collagen and elastic fibers in the reticular dermis, which is associated with skin wrinkle formation.^1^, ^2^, ^6^, ^7^, ^8^ Note that *β*‐NAG2 can be used as UDP‐NAG for glycosylation of proteins with *o*‐linked NAG and/or synthesis of glycoconjugates, for example, lumican (keratan sulfate proteoglycan) and decorin (dermatan/chondroitin sulfate proteoglycan).^34^ Of interest, previous studies reported that *o*‐linked NAG modification stimulates collagen synthesis in cardiac fibroblasts,^35^ and lumican and decorin play important roles in collagen fibrillogenesis.^34^, ^36^ Importantly, HA interacts with collagen and elastic fibers in the dermis,^37^ and HA‐mediated skin hydration can affect the recoil capacity and tensile strength of these fibers. For better understanding of antiwrinkle activity of *β*‐NAG2, further detailed analyses are needed to investigate effects of *β*‐NAG2 on production of collagen and/or glycoconjugates in dermal fibroblasts and/or interaction of HA with collagen and elastic fibers in the papillary and reticular dermis of *β*‐NAG2‐treated skin.

In conclusion, we have shown from in vitro and in vivo studies that *β*‐NAG2 possesses antiwrinkle activity, and propose the possibility that the promotion of epidermal HA production by application of cosmetics or ointment containing *β*‐NAG2 would be a useful remedy to improve or prevent aging symptoms.

## CONFLICT OF INTEREST

The authors declare that there is no conflict of interest.
